# Optimization of Process Parameters for a Chemi-Absorbed Graphene Coating and Its Nano Tribological Investigation

**DOI:** 10.3390/nano10010055

**Published:** 2019-12-25

**Authors:** Pengfei Li, Yuncheng Li, Hongyue Chen, Hui Liu, Xianhua Cheng

**Affiliations:** 1 School of Mechanical Engineering, Liaoning Technical University, Fuxin 123000, Chinachyxiaobao@126.com (H.C.); lauhwi@163.com (H.L.); 2 School of Mechanical Engineering, Shanghai Jiao Tong University, Shanghai 200240, China

**Keywords:** graphene surface coating, nano-tribology, process optimization, self-assembly, hydrothermal reduction

## Abstract

A reduced graphene oxide coating was deposited on a titanium substrate for potential anti-friction applications in nano- or micro-mechanical systems. A γ-aminopropyltriethoxysilane coating was self-assembled on the substrate as an adhesive interlayer beforehand. The process parameters of self-assembly and hydrothermal reduction of graphene oxide coating were explored via water contact angle and tribological tests. Insufficient self-assembly duration of graphene oxide layer can be detected by water contact angle results, and the corresponding coating displayed a higher coefficient of friction and shorter anti-wear lifetime than the optimized one. Proper hydrothermal temperature and duration were also confirmed by its water contact angle, coefficient of friction and anti-wear lifetime. Noticeably, excessive hydrothermal temperature or duration would reduce the coefficient of friction, but diminish the anti-wear resistance. The optimized process parameters were confirmed as assembly duration of graphene oxide coating for 12 h, hydrothermal reduction duration of 6–8 h at 135 °C. Nano tribological behaviors of the obtained hydrothermal reduced graphene oxide coating by AFM tester were then investigated under various testing circumstances. The results showed that the coating performed reliable and low adhesion and friction forces under all circumstances. The nanowear resistance of the titanium substrate was significantly strengthened by the prepared coating.

## 1. Introduction

Since its discovery in 2004 [1], graphene has been considered as an important two-dimensional nanomaterial in electronic and mechanical fields. Owing to its high thermal conductivity [2], low surface energy [3] and large in-plane elastic modulus [4], graphene displays outstanding frictional and wear resistant behaviors on the micro [5,6,7] and nano [8,9,10] scales. Most frequently, graphene is used as a physically absorbed solid lubricating coating [11] or additive material for lubricants [12,13]. Graphene sheets are often coated to the contact surface of parts (i.e., substrates) by physical deposition methods such as electrostatic adsorption [14] and in-situ growth [15]. The nano-tribological properties of those coatings are usually researched under ultra-low applied loads [11]. When the applied load is raised to a certain extent, those physically adsorbed graphene coatings are apt to experience wear failure owing to poor adhesion with the substrate [16]. The tribological properties of graphene coatings have also been shown to depend on their interactions with the underlying substrate surface [17,18].

Thus, methods of enhancing the adhesion between graphene coating and substrate must be explored to enhance the wear resistance. A chemically absorbed graphene coating has been prepared on titanium substrates in this study. Owing to the inertness of graphene material, a graphene oxide (GO) intermediate material is used at first to allow chemical bonding to the substrate and then hydrothermally reduced to obtain a reduced graphene oxide coating. For constructing a stronger coating-substrate interface bonding, 3-aminopropyltriethoxysilane (APS), which has chemically active head groups at both ends of its molecule after hydrolysis, has been proved to have the capacity of chemically attaching graphene oxide sheets onto the substrate by the self-assembly approach [19]. In our previous study [20], the self-assembly process of APS coating has already been optimized for the purpose of enhancing tribological performances of the graphene coating.

Water contact angle (WCA) determination is a popular and accessible detection method for the surface wettability of coatings [21,22]. APS, GO and reduced GO coatings have different WCA values [19]. Thus, WCA can be applied to verify the completion of GO assembly and reduction. The resultant coefficient of friction and anti-wear lifetime obtained in micro tribological tests are proved to indirectly clarify the surface property and interlayer bonding strength of coatings [19,20,23] and thus can be utilized as a criterion for optimization of GO coating assembly and reduction processes.

In this study, the self-assembly and hydrothermal reduction processes of GO coatings have been discussed in details by means of WCA and microtribological investigation. After optimization of the coating preparation, the adhesion and friction properties of HRGO-APS coating on titanium substrate in nanoscale has been investigated under varied circumstances. Additionally, the nanowear properties of the prepared coatings and the titanium substrate were also studied.

## 2. Materials and Methods

### 2.1. Pretreatment of Ti Substrates

A thickness of ~150 nm titanium (99.8%) material was DC sputter-coated in high vacuum onto polished SiO_2_ plates (approximately 10 mm × 10 mm × 0.7 mm) for AFM morphology and nanotribological tests (called henceforth deposited Ti substrate). Considering that the deposited Ti layer has a weak bonding with the SiO_2_ substrate, TNTZ (Ti–29Nb–13Ta–4.6Zr) alloy was utilized as Ti substrates for microtribological tests. The TNTZ alloy plates were cut to a size of 10 mm × 10 mm × 1 mm and mechanically polished. That plate is named as titanium alloy substrate. The substrates were steeped into 1 M sodium hydroxide aqueous solution for hydroxylation before coating deposition.

### 2.2. Deposition Process of Self-Assembled Coatings

The self-assembled APS coatings on deposited Ti substrate and TNTZ substrate were prepared according to our previous work [24], in order to enhance the interface bonding strength between graphene coating and the titanium substrate. Pure graphene is too inert to be chemically deposited onto titanium substrates. Graphene oxide (GO) was thus utilized to form a GO layer by the self-assembly method, and the corresponding sample is named as GO-APS coating. Then the oxygen groups on the surface of the GO coating were reduced to improve its hydrophobicity by hydrothermal reduction treatment. After the reduction treatment, the obtained sample was named as HRGO-APS coating. Thus in this study, the graphene coating was in the form of reduced graphene oxide layer. 

The self-assembly of GO layer was as follows: an appropriate amount of GO powder obtained by a modified Hummers method [19] was dropped into deionized water with a weight ratio of 5:10,000. After an ultra-sonication treatment for 30 min, GO colloidal solution was obtained. Then deposited Ti substrates or TNTZ substrates were kept in the GO colloidal solution at 60°C for various durations from 2 h to 24 h.

The hydrothermal reduction of GO coating was as follows: samples of GO-APS coatings were put into a Teflon-liner which was filled with deionized water. That liner was then put inside a stainless steel autoclave, and heated at different temperatures for various durations in a heating furnace.

### 2.3. Testing Procedure

Before testing, samples were dried with an electric blower to keep the sample surface clean and free of large particle contamination. The water contact angle (WCA) of each coating were measured by DSA100 optima (KRUSS, Hamburg, Germany) system. The water drops on the sample surface were about 0.6 μL each time. For each sample, more than five points at different positions were determined during the WCA test and the average value was recorded as its final WCA value. 

A universal micro-tribotester (UMT-2, CETR, Madison, WI, USA) was used to test the friction and wear properties of the coatings on titanium alloy substrate in the ball-on-disc mode. Si_3_N_4_ balls (diameter of 3 mm, mean roughness less than 0.02 μm) were used as the contact ball, running at a sliding distance of 5 mm per pass and a sliding frequency of 1 Hz, under an applied load of 100 mN. All UMT-2 tests ran at room temperature (20 °C) and with relative humidity between 20–30%. The coefficient of friction (COF) was obtained from the COF vs time curve before the COF value suddenly raised to a much higher level, which indicated that the coating was worn out. Average of COF data before sudden rise in one curve was considered as COF of the curve. The COF value of a sample was the average value of the COF data obtained from three friction test curves of that sample. The value of anti-wear lifetime was approximately recorded according to the COF vs time curve before the coating was worn out. The average value of the three measured values was considered as the anti-wear lifetime value for a sample.

An atomic force microscope (AFM, Nanoman VS, Veeco, Plainview, NY, USA) was applied for surface morphology investigation and nanotribological measurements. Nanotribological measurements were performed under contact mode by using square pyramidal Si tips with nominal tip radius of 30–50 nm, the spring constant of mounted on gold-coated triangular Si cantilevers was about 0.67 N/m. Colloidal tips for AFM nano wear test were custom built. Each silica spherical head with diameter of ~30 μm was mounted on a tipless silicon cantilever. The colloidal tips had the normal stiffness of ~4 N/m and the torsional spring constant of 1.66 × 10^−8^ N·m.

## 3. Results

### 3.1. Determination on Length of Time for GO Assembly

**Figure 1:**
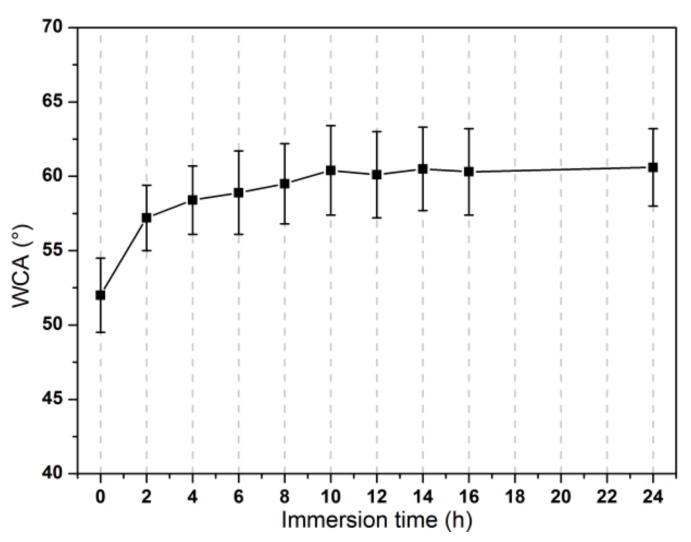
The influence of immersion time of GO assembly upon WCA values for GO-APS coating.

The assembly process of graphene oxide sheets on as-deposited APS coating on Ti substrate was discussed in this section. Samples of APS coating were immersed into GO aqueous solution (with ~0.05 wt.% of GO material) at 60 °C for varied assembly time: 0 h, 2 h, 4 h, 6 h, 8 h, 10 h, 12 h, 14 h, 16 h and 24 h. The variation of WCA values at different assembly durations for GO-APS coatings were shown in Figure 1.

It can be seen from Figure 1 that the WCA values of GO-APS coatings increased from 53° (±3.4°) at 0 h to 60.1° (±2.9°) at 12 h as the self-assembly duration increases. At 0 h, the surface of the sample should be just APS coating, and thus the WCA value (52.0°) presented the same WCA value as APS coating [19]. In the first two hours of the assembly, the WCA value of the film increased rapidly. During the assembly duration from 2 h to 10 h, the increase of WCA value slowed down. Those phenomenon indicated that: the assembly efficiency of GO materials was very high during the first two hours and a large number of GO sheets were rapidly adsorbed on the surface of the sample. As the surface of APS coating was gradually covered by more and more GO sheets, the area of the exposed APS material decreased, so that the assembly efficiency of GO materials decreased obviously. In Figure 1, the WCA values from 10 h to 24 h were approximately the same, indicating that GO sheets have fully covered the substrate surface. Thus, the assembly time of GO coating should be more than 10 hours.

**Figure 2:**
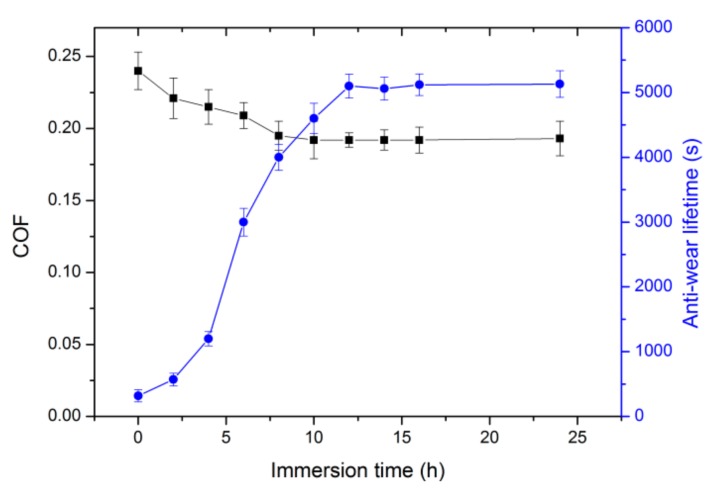
The influence of immersion time of GO on COF and Anti-wear lifetime of GO-APS coating on TNTZ alloy substrates.

The change of COF and anti-wear lifetime of GO-APS coating with various assembly time are presented in Figure 2. Data at 0 h corresponds to the frictional data of APS coating. Generally, the anti-wear lifetime increased with the increase of GO assembly time, and the COF decreased with the increase of GO assembly time. According to the WCA results in Figure 1, a large number of GO sheets were adsorbed on the surface of APS coating at 2 h, so the COF of the coating was significantly reduced from 0.24 at 0 h to 0.22 at 2 h. Since there were GO and APS materials exposed on the surface of the sample at the same time at 2 h, the COF value was between the values of GO and APS coatings. The corresponding anti-wear lifetime of that GO-APS coating was as short as 500 s. The poor wear resistance of that GO-APS coating was attributed to the weak interlayer binding force between APS and GO.

As the assembly time increased, on the one hand, more GO sheets were covered on the surface of the sample, and the corresponding COF of the sample was gradually approaching to the value of GO coating (~0.19, as the COF value after 12 h in Figure 2); on the other hand, the active oxygen-containing group of GO molecule gradually reacted with the amino group of APS coating by forming a chemical bond, which improves the interlayer bonding force of the coating and thus the anti-wear lifetime value increased. At assembly time of 12 h, the COF and the anti-wear lifetime reached to the best state, indicating that GO molecule had completely covered the surface of APS coating, and the interlayer chemical bonding was the strongest. Thus, GO-APS coating could be successfully assembled by 12 h.

Noticeably, after an assembly time of 10 h, the COF value reached a steady stage (as can be seen in Figure 2). However, its anti-wear lifetime was just about 4600 s, much lower than that of samples with assembly time of more than 12 h. It is assumed that GO sheets could be fully covered on the surface of the sample for assembly time of 10 h, but the chemical bonding between APS and GO layers was not finished. After the assembly time of 12 h, both anti-wear lifetime value and COF value reached to a steady stage. Thus, the optimal assembly time of GO coating should be 12 h.

### 3.2. Determination of Temperature and Time during Hydrothermal Reduction Process

After the confirmation of GO assembly duration, the parameters of temperature and time in the followed process of hydrothermal reduction were discussed to investigate their influences on the tribological properties of HRGO-APS coating and to optimize those process parameters.

**Figure 3:**
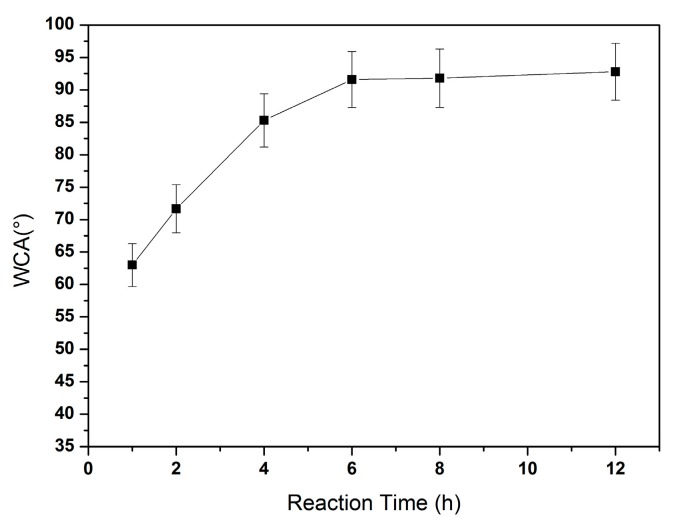
The influence of reaction time for hydrothermal reduction upon WCA values for HRGO-APS SAM.

First, lengths of hydrothermal time (reaction duration) were analyzed. It was set as 1 h, 2 h, 4 h, 6 h, 8 h and 12 h, at temperature of ~135 °C. Under a higher temperature of 150 °C, the resultant data was similar and thus not presented here. The WCA test results of the corresponding HRGO-APS coatings were shown in Figure 3. Generally, the higher the WCA value of a coating, the better the hydrophobicity of its surface is, which means a better reduction efficiency on the oxygen-containing functional groups of GO coating. When the reduction reaction time was 1 h, the WCA of the coating was about 63.0° ± 3.3°, higher than that of untreated GO coating (60.1° ± 2.9°), indicating that the hydrothermal method effectively reduced the GO coating within the first hour. When the reaction time reached to 6 h, the WCA value was about 91.6°. The WCA of the coating had no obvious change when the reaction time extended to 8 h, 10 h and 12 h. The results of WCA data showed that the reaction time of hydrothermal method should be more than 6 h.

**Figure 4:**
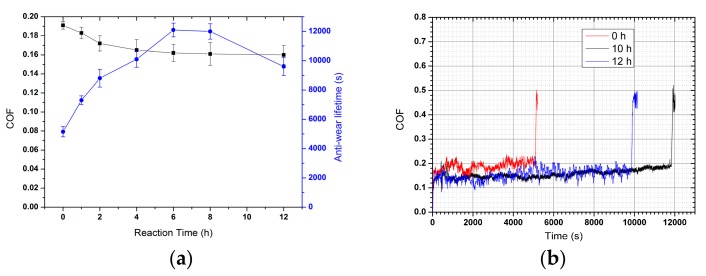
The influence of reaction time for hydrothermal reduction on COF and Anti-wear lifetime of HRGO-APS coating on TNTZ alloy substrates (**a**); COF vs time curves of coatings with three different reaction times—0 h (GO-APS coating), 10 h and 12 h (**b**).

The influence of different hydrothermal method reaction time on the COF and anti-wear lifetime of HRGO-APS coating is shown in Figure 4a and the COF vs time curve of three typical coatings is presented in Figure 4b. With the increase of reaction time, the COF decreased and the anti-wear lifetime increased as seen in Figure 4a. When the reaction time of hydrothermal reduction was set at 12 h, the COF of value was not significantly changed compared with that at 8 h. However, the anti-wear lifetime was significantly reduced. There COF vs time curves are presented in Figure 4b. Even though those two curves had similar COF values, the curve of 12 h experienced more fluctuation than the curve of 10 h did. It is assumed that 12 h of hydrothermal treatment was so long that the interlayer bonding force was harmfully affected. Thus graphene sheets were peeled off as the tribological test continued, which induced energy dissipation and fluctuation of COF value. The above analysis showed that the optimal reaction time of hydrothermal method should be 6–8 h. As a comparison, the COF vs time curve of GO-APS coating was also showed in Figure 4b. The COF of GO material was higher than that of HRGO and its wear resistance was weaker.

The influence of hydrothermal reaction temperature on the tribological performances of HRGO-APS coatings was also analyzed. According to [25], the effective reaction temperature of hydrothermal reduction on graphene oxide can be ranged from 120 °C to 180 °C. The parameter of reduction temperature could have an influence on the reduction effect and the wear resistance of the coating. Thus, reaction temperatures of hydrothermal reduction were varied from 80 °C, 100 °C, 120 °C, 135 °C, 150 °C, 160 °C and 180 °C, with reaction time of 7 h.

**Figure 5:**
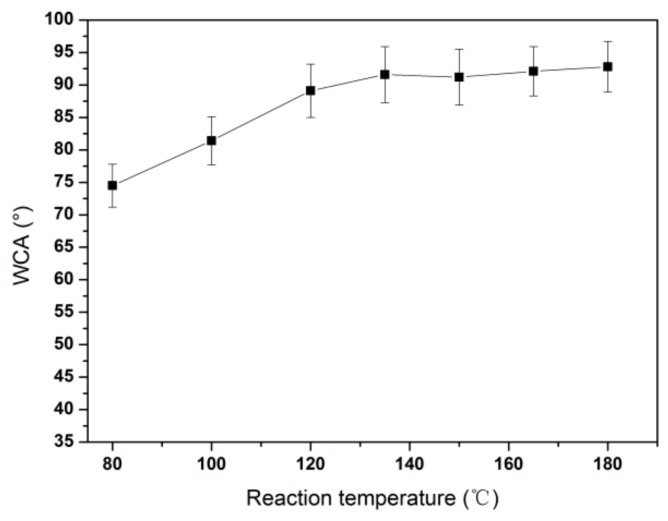
The influence of reaction temperature for hydrothermal reduction upon WCA values for HRGO-APS coating.

The WCA results of those coatings were shown in Figure 5. The WCA results showed that the surface wettability of GO-APS was greatly affected by hydrothermal reduction at all seven temperatures. WCA values of the coatings were risen from 74.5° at 80 °C to 89.1° at 120 °C. There was no significant difference in WCA value when the temperature was higher than 120 °C, indicating that the process could significantly improve the hydrophobicity of the coatings as long as the temperature was above 120 °C.

**Figure 6:**
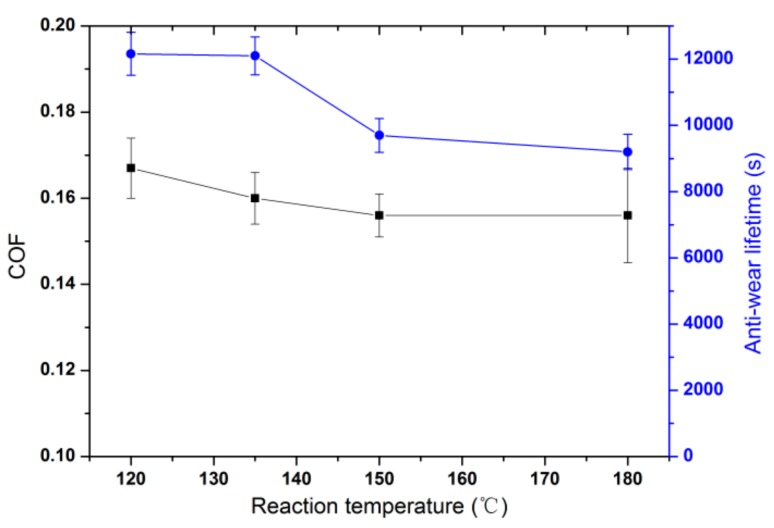
The influence of reaction temperature for hydrothermal method on COF and anti-wear lifetime of HRGO-APS coating on titanium alloy substrates.

The tribological tests of HRGO-APS coating obtained at hydrothermal reduction temperatures of 120 °C, 135 °C, 150 °C and 180 °C with a reaction time of 7 h were investigated and the resulting COF and anti-wear lifetime values are shown in Figure 6. There was no significant difference in the COF values of the coatings under all four cases (from 0.167 at 120 °C to 0.156 at 180 °C). When the temperature was set as 150 °C, the anti-wear lifetime of the coatings decreased obviously compared with that at 135 °C. When the reduction temperature reached to 180 °C, the anti-wear lifetime of the coatings decreased to less than 10000s. Thus, hydrothermal reduction temperature should be lower than 150 °C to avoid a decrease of wear resistance for the HRGO-APS coating.

**Figure 7:**
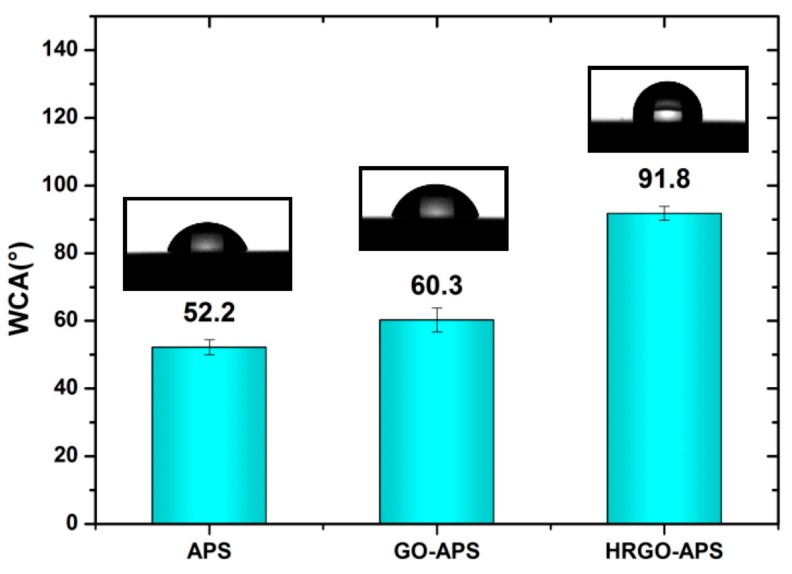
WCA data of hydroxylated APS coating, GO-APS coating and HRGO-APS coating (Insets are images of water droplets on tested surface).

Therefore, the hydrothermal reduction process was optimized as the reaction time of 6–8 h, and temperature of 135 °C. The WCA values of as-obtained APS coating, GO-APS coating and HRGO-APS coating after process optimization were illustrated in Figure 7. Both WCA values of APS and GO-APS self-assembled coatings were less than 90°, which were hydrophilic. After reduction by hydrothermal method, the WCA value of the coating increased to ~90°, indicating that the surface energy of the coating decreased significantly.

### 3.3. Surface Morphologies of the Self-Assembled Coating

**Figure 8:**
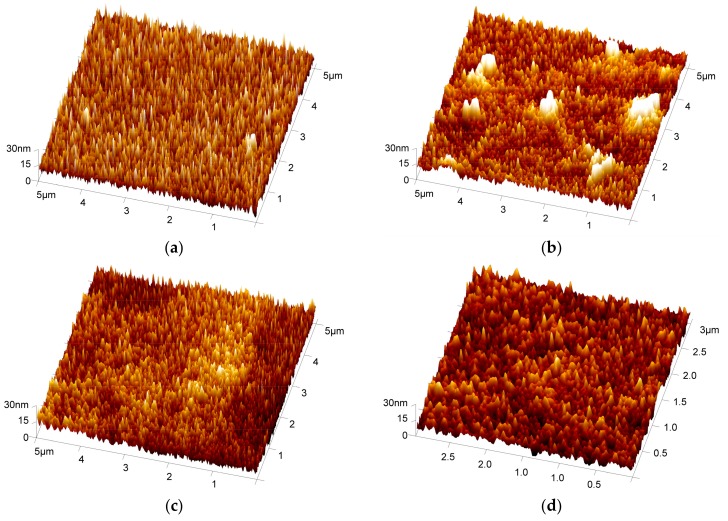
AFM 3D morphology tests for (**a**) deposited Ti substrate, (**b**) GO-APS, (**c**) HRGO-APS (5 μm × 5 μm), and (**d**) HRGO-APS coating on the deposited Ti substrate (3 μm × 3 μm).

The AFM morphological graphs of obtained GO and HRGO-APS coatings on deposited Ti substrate were shown in Figure 8b–d. For comparison, surface morphology of the substrate was also detected (shown in Figure 8a). Theoretically, the surface shape of deposited Ti substrate by DC spraying should be spherical particles on the top. However, there is a deviation in the shape scanning of this spherical object for AFM instruments, and thus the obtained AFM surface morphology of that substrate was sharp particles. The height of those sharp particles were 15 nm approximately, detect by the section function of AFM analysis software --NanoScope Analysis 1.40. When the HRGO-APS coating was successfully assembled on the surface of the substrate, the morphology was obviously smoothed, as shown in Figure 8c,d. The surface roughness of the deposited Ti sample and the HRGO-APS can be obtained from Figure 8a,c by AFM analysis software (Nanoscope Analysis, version 1.40) as 5.53 nm (Ra) and 6.97 nm (Rq) for deposited Ti substrate, and 4.91 nm (Ra) and 6.14 nm (Rq) for HRGO-APS coating. Because of the coverage of graphene sheets, the surface was smoothed. The thin HRGO-APS coating should be so tightly attached to the substrate that the shape of the spherical particles of the substrate under HRGO-APS coating was still vaguely visible. Thus, surface roughness value of HRGO-APS coating was only a little lower than that of the substrate.

The surface morphology of GO-APS coating was presented in Figure 8b. Though most of the area was smoother than deposited Ti substrate, there were several large and high islands which were presented as bright spots. Thus, the surface roughness was raised to Ra = 5.52 nm and Rq = 8.71 nm. The emergence of islands and increase of surface roughness were attributed to contamination absorbing, in accordance with previous research about GO coatings [19].

## 4. Nano-Tribological Performances of Prepared Poating

By the analysis of Section 3, the manufacturing process of HRGO-APS coating has been optimized. However, before the application of this coating in Nano Electro-Mechanical System (NEMS), nanotribological properties of this coating under varied circumstances should be investigated.

In this section, the nanotribological performances of HRGO-APS coating obtained after process optimization were investigated to evaluate the application perspective of HRGO-APS coating in NEMS. For comparison, nanotribological properties of GO-APS coating, APS coating and deposited Ti substrate were also presented. Adhesion forces were measured at tapping mode using force calibration plot and the results were obtained by the same method with ref. [26]. Friction forces were tested at contact mode at a rectangle area of 2 μm × 200 nm, approximately. The mean value of forward and backward scanning voltage signals during friction force test was obtained and converted to force units (nN) according to a simple calibration [27].

**Figure 9:**
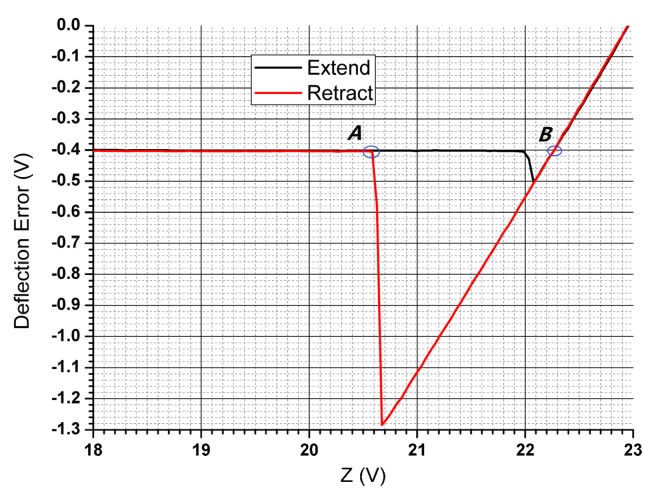
Typical data of deflection Error vs Z displacement curve obtained in adhesion force test.

In detail, adhesion force was determined according to the force calibration plot (deflection Error vs Z displacement curve) in Figure 9, which was obtained by AFM nanotribological test. The Z difference value of point A and B was coded as Δ*Z.* Then adhesion force can be calculated by Equation (1) below:

*F_ad_* = *S_c_* × *S_d_* × Δ*Z* (1)

where *S*_*c*_ (N/m) is the spring constant of the probe tip, and *S* (nm/V) is the deflection sensitivity of the equipment. Sensitivity can be obtained via the data file of the test by NanoScope Analysis 1.40, which was 32.91 nm/V.

### 4.1. Adhesion and Frictional Forces under Different Conditions

Before the adhesion test, the probe scanned the surface morphology of the sample in contact mode to wear out the sharp end of the AFM probe tip. By doing this, the stability of the diameter of the probe tip can be guaranteed as much as possible. Furthermore, the pre-test scanning can promote the tribochemical reaction of the AFM tip surface, so that the chemical state can be close to the state of friction force test.

#### 4.1.1. Adhesion and Frictional Forces under Various Applied Loads

**Figure 10:**
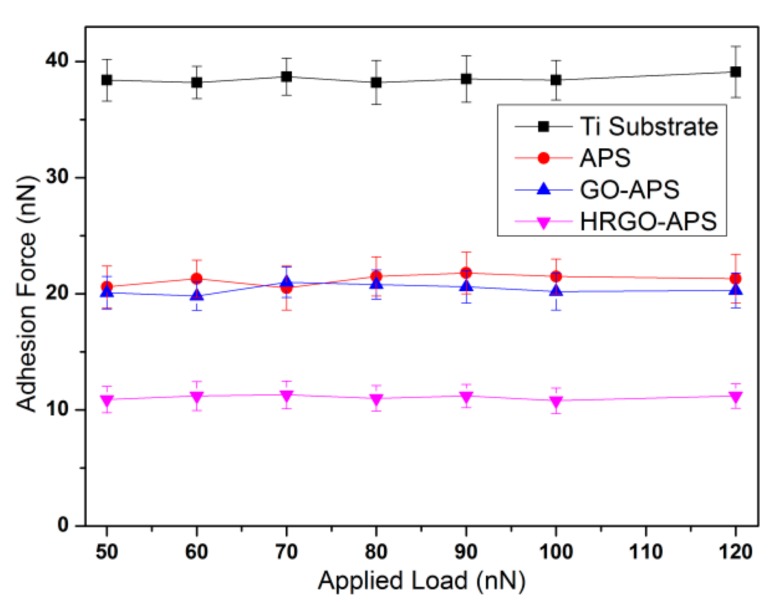
Variation of the adhesion force of Ti substrate and prepared coatings with the change of applied load (with scanning rate of 2 Hz).

The adhesion force results (with scanning speed of 2 Hz, at relative humidity RH of about 30% and temperature of 22–25 °C) of each sample are shown in Figure 10. The adhesion forces of each sample were not obviously affected by the applied load, among which the adhesion force of Ti substrate was the largest and HRGO-APS coating was the lowest. The adhesion forces of APS and GO-APS coatings were very close. Those results were consistent with the WCA results of each sample. It is apparent that the surface wettability of samples is a very important factor in affecting the adhesion force.

The adhesion between the sample surface and the probe tip are usually the combination of capillary force F_C_, van der Waals force F_V_, electrostatic adsorption force F_E_ and interface bonding force F_B_, which is:

Fa_d_ = F_C_ + F_V_ + F_E_ + F_B_ (2)

In this study, both the probe and the sample were placed in the air for a period of time, and both the surfaces did not absorb external charges, which means F_E_ = 0; In the short period of contact between the probe and the sample during adhesion test, it should not occur any tribochemical reaction, which means F_C_ = 0; Van der Waals force F_V_ is related to the distance between two surfaces but not related to the load applied in the sample, so F_V_ remains unchanged for the same sample; Capillary force F_C_ are usually affected by WCA of the surface, and thus F_V_ should not change for the same sample with different applied load. Therefore, adhesion force of each sample should not change significantly with the increase of applied load.

**Figure 11:**
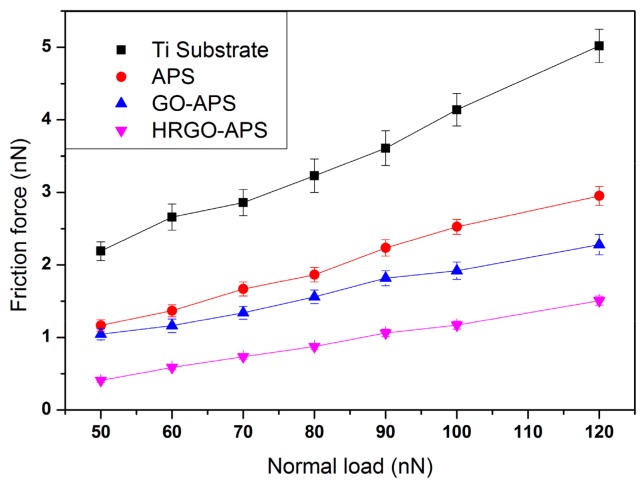
Variation of friction forces of Ti substrate and prepared coatings with the change of applied loads (with scan rate of 2 Hz; scan area: 2 μm × 2 μm).

Figure 11 presented the friction force results of those four samples (with scanning speed of 2 Hz, and at relative humidity of about 30%). It can be seen that the friction force of the four samples increased linearly with the increase of applied load from 50 nN to 120 nN. Under the same applied load, the friction force of HRGO-APS coating was the smallest, and that of deposited Ti substrate was the largest.

The friction of the four coatings increased with the increase of the applied load, which was attributed to the increase of the contact area between probe tip and the coating. According to Homola [28], the friction of adhesive contact surfaces in nano scale should be interface friction, in which the friction force F_fr_ should consist of three terms:

F_fr_ = C_1_F_ap_^2/3^ + C_2_F_ap_ + C_3_F_a__p_^4/3^ (3)

where F_ap_ is the applied load, C_1_, C_2_ and C_3_ are the coefficients related to adhesion energy, elastic coefficient and contact radius, respectively. When F_ap_ is relatively high, the second term dominates F_fr_. In that case, the calculation of friction force is similar to the traditional calculation formula of friction force: F_fr_ = µF_ap_.

In our test, the applied load was as low as under 120 nN, the third term of Equation (3) can be neglected [28]. Thus, the difference of friction force between different coatings under the same applied load should be attributed to the adhesion forces during their contact with the probe tips, and the increase trend of friction force for the same coating is attributed to the increased contact surface as the applied load increases.

According to Figure 10 and Figure 11, all three kinds of prepared coatings improved the nanotribological properties of the substrate to varied extend, among which HRGO-APS coating behaved the best. GO-APS and APS coatings had similar adhesion force values, but GO-APS coating had lower friction forces under all applied loads. That is attributed to the structural difference of those two coatings.

According to Frisbe [29], when two contact surfaces with ending group of carboxyl (-COOH) or alkyl group (CH_x_) in a nanotribological test, the adhesion force of *F_COOH/CHx_* is the lowest, *F_COOH/COOH_* is the highest and *F_CHx/CHx_* was intermediate. According to Opitz [30], when the silicon probe sliding on the surface of tested sample, it is easy to generate tribo-chemical reaction, which makes the silicon probe hydroxylated form silicon hydroxyl groups (Si-OH) on the surface. In this study, hydroxyl group was considered as the similar effect with carboxyl group on adhesion force. When the probe modified with hydroxyl group contacted with the active groups on the surface of the sample, such as hydroxyl and carboxyl, adhesion was in the state of *F_OH/OH_* or *F_OH/COOH_*. However, when the probe contacted with the surface without active groups, the adhesion state was *F_OH/CHx_*, which was lower than the former case. This theory can be used to explain the difference of adhesion forces and friction forces between GO-APS and HRGO-APS coatings. The surface of HRGO-APS coating contained less active groups than GO-APS did by hydrothermal reduction treatment. Thus, during the test, F_OH/OH_ or *F_OH/COOH_* dominated the adhesion state of GO-APS against hydroxylated Si probe tip, while there should be more F_OH/CHx_ in the case of HRGO-APS. That was the reason that HRGO-APS coating held a lower adhesion force than GO-APS did. Adhesion plays an important role in affecting the value of friction force. In general, the greater adhesion is, the greater the friction force. Thus, the friction force of HRGO-APS was lower than that of GO-APS coating in Figure 11.

#### 4.1.2. Adhesion and Frictional Forces under Various Scanning Speeds

At the temperature of 22–25 °C, relative humidity of ~30%, and applied load of 100 nN, the adhesion and friction forces of different samples were tested under various sliding speeds. The scanning speed was set to be at the range of 20–500 μm/s with scanning of 5 μm (that was the scanning frequency of 2–50 Hz). In this section of the adhesion test, the adhesion force was determined during the friction force test when the applied load was 0 nN, the same method with ref. [31].

**Figure 12:**
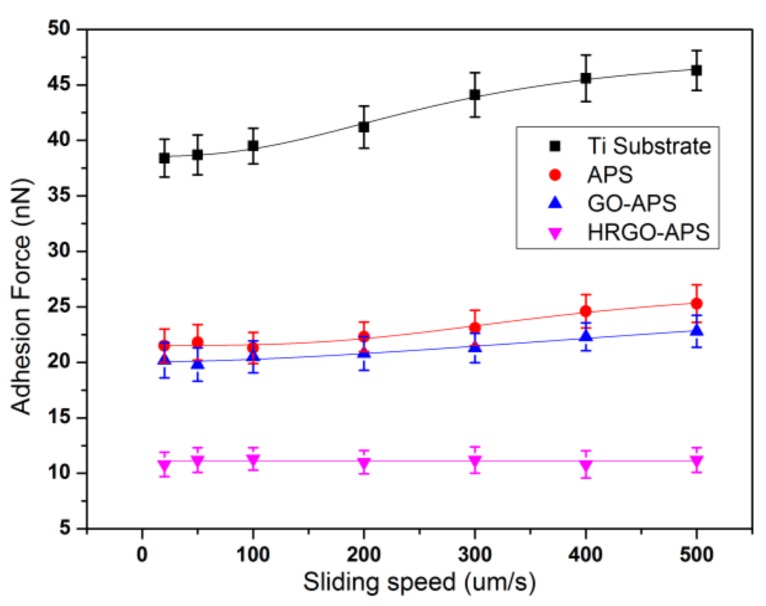
Variation of the adhesion force of Ti substrate and prepared coatings with the change of sliding speed (applied load: 100 nN, Scan area: 2 μm × 2 μm).

The influence of scanning speed on adhesion forces of four kinds of samples was shown in Figure 12. At each scanning speeds, HRGO-APS coatings performed the lowest adhesion force and deposited Ti substrate behaved the highest.

According to the nonlinear change trend of the adhesion force, a logistic fitting method was implemented to fit the data of Ti substrate, APS coating and GO-APS coating, by Origin 8.5.1. The fitted curves correctly presented the change rule of the original data as can be seen in Figure 12. The fitting results is consistent with the theory of Bhushan [31]. The fitting equation is shown as Equation (4):

y = A_2_ + (A_1_ − A_2_)/(1 + (x/x_0_)^p^) (4)

Variables of A_1_, A_2_, x_0_ and p for each sample were presented in Table 1.

The adhesion vs scanning velocity curve of Ti substrate showed an apparent logistic relationship. When the scanning speed was low, the slope increase of the adhesion curve was small. With the increase of scanning speed, that slope also increased. When the scanning speed reached to 400–500 μm/s (40–50 Hz), the slope increase of the adhesion force curve decreased. The adhesion vs scanning speed curves of APS and GO-APS coatings also conformed to this rule. However, the adhesion force of HRGO-APS coatings was almost unaffected by scanning speed.

**Figure 13:**
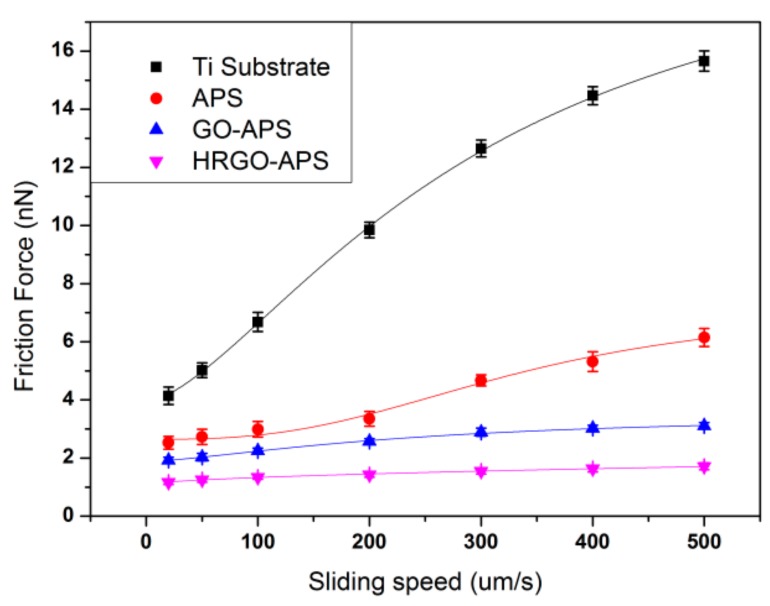
Variation of the friction force of Ti substrate and prepared coatings with the change of sliding speed (applied load: 100 nN; scan area: 2 μm × 2 μm).

Figure 13 showed the friction force variation of each sample at different scanning speeds. The friction force of Ti substrate and self-assembled coatings increased with the increase of sliding speed. The friction force curve of Ti substrate experienced the most increment, while that of HRGO-APS coating presented the least. At all tested sliding speeds, HRGO-APS coating had the least friction forces, which was attributed to the following two factors: (1) HRGO-APS coating had the lowest adhesion force (as shown in Figure 12), so that the least dissipation energy was consumed during the slide of the probe tip; (2) the intrinsic anti-friction property of graphene material was better than that of titanium and APS material. The curves in Figure 13 were fitted by the same method as Figure 12. Variables are listed in Table 2.

The adhesion and friction forces of the samples increased as scanning speed increased, indicating that the frictional work between the interfaces increased with the increase of scanning speed. Nevertheless, the increase trend was not linear and the increase slopes during the early and final stages were smaller than at their medium stage. That was attributed to the influence of meniscus force. At early stage when scanning speed was low, water molecules in the air can be absorbed onto the surface of sample and probe tip. For hydrophilic surfaces, the absorbed water was enough for the formation of meniscus. Owing to the meniscus effect, the movement of probe tip was inhibited to certain extend [31,32]. The smaller the scanning speed was, the easier the water molecules formed the meniscus, which made the value of adhesion and friction relatively higher in the early stage; with the increase of scanning speed, the formation of meniscus became harder and harder, and the influence of meniscus force on adhesion and friction forces was reduced at medium and final stages. As the scanning speed increased to the final stage (fastest), the work of friction increased, but the surface of sample of probe tip were assumed to experience wear which consumed part of the energy, and thus the increase slopes of adhesion and friction forces was lower down.

#### 4.1.3. Adhesion and Frictional Forces under Various Relative Humidity

**Figure 14:**
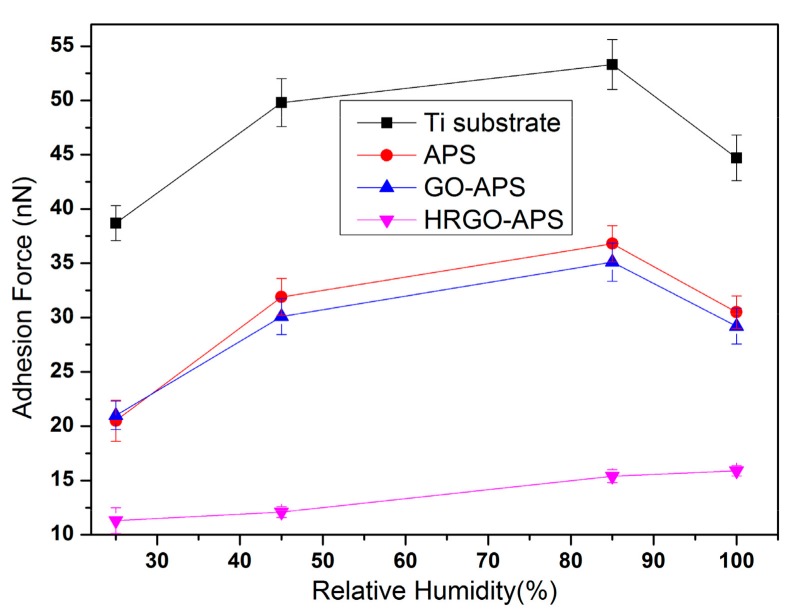
Variation of adhesion forces of deposited Ti substrate and prepared coatings under various RHs (Applied Load: 70 nN).

**Figure 15:**
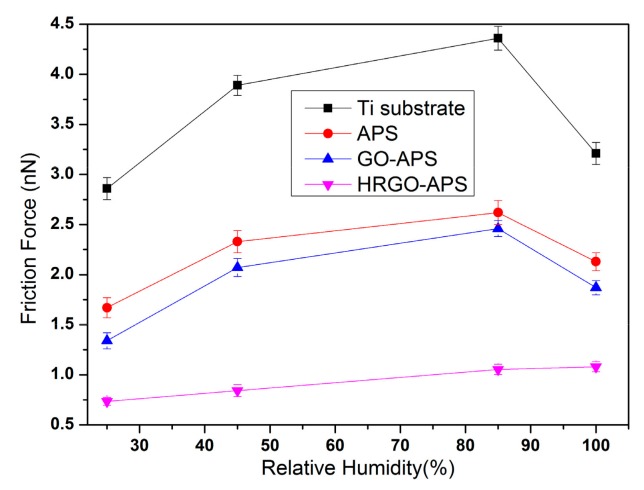
Variation of friction forces of deposited Ti substrate and prepared coatings under various RHs (Applied Load: 70 nN, Scan area: 2 μm × 2 μm, Scan rate: 2 Hz).

The nanotribological properties of the self-assembled coatings are closely related to the wettability of their surface. Under the condition of high relative humidity (RH), water molecules can be absorbed onto the surface of the sample, which can affect the adhesion and friction force results to some extent. Therefore, it is necessary to investigate the nanotribology of the samples under different RH values. Adhesion and friction force tests were implemented under four RH values (approximately 25%, 45%, 80% and 100%). RH was adjusted by an air humidifier and an air conditioner. The tested samples were kept under the aimed HR value before test. During the tests, experimental temperature was kept in 22–25 °C and the applied load was 70 nN. The adhesion and friction force results of deposited Ti substrate and prepared APS, GO-APS and HRGO-APS coatings were shown in Figure 14 and Figure 15.

Adhesion and friction forces increased as RH increased when RH was under 85% for all tested samples. As the increase of RH, more water molecules could be absorbed onto the interface between tested surface and probe tip, so that the meniscus effect was enhanced. Thus, the adhesion and friction forces of those four samples increased.

When RH reached to 100%, a thick layer of water molecules was assumed to be adsorbed onto the surface of hydrophilic samples (Ti substrate, APS coating and GO-APS coating). Thus the meniscus effect disappeared and the water layer acted as boundary lubricating film between the tested surface and the probe tip. However, for hydrophobic surface of HRGO-APS coating, absorbed water was not enough to form a lubricating film. Therefore, its adhesion and friction forces still increased as RH raised from 80% to 100%. From RH 25% to RH 100%, the adhesion force increased from 11.3 to 15.9 and the friction force increased from 0.73 to 1.08 in the case of HRGO-APS coating. The performance of HRGO-APS coating was proved to be much lower and more reliable than that of Ti substrate.

#### 4.1.4. Adhesion and Frictional Forces under Various Temperatures

**Figure 16:**
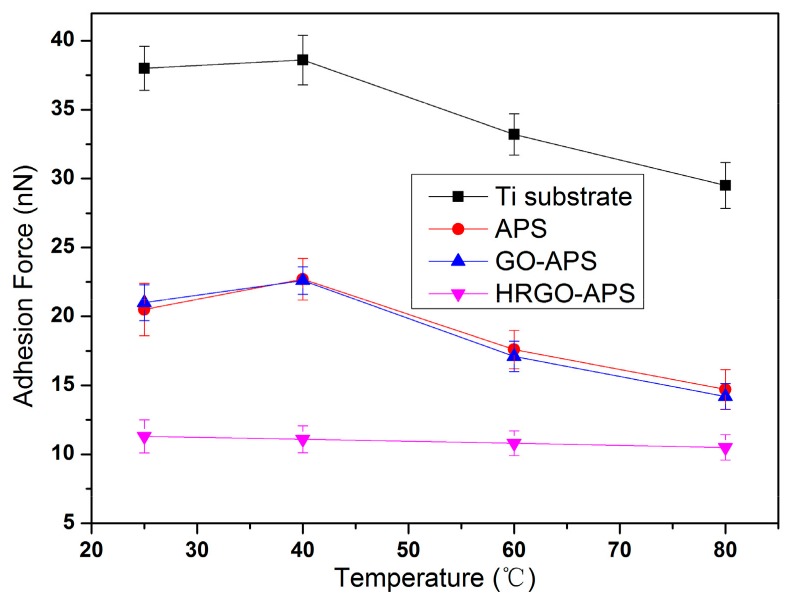
Variation of adhesion forces of hydroxylated Ti substrate and prepared coatings under various Temperature (Applied Load: 70 nN).

**Figure 17:**
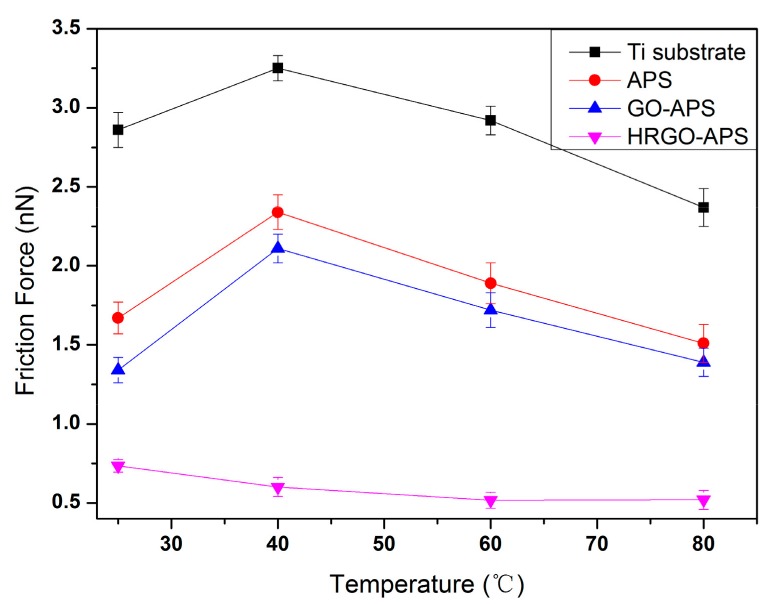
Variation of friction forces of hydroxylated Ti substrate and prepared coatings under various temperatures (applied load: 70nN; Scan area: 2 μm × 2 μm; scan speed: 2 Hz).

The adhesion force results (under applied load of 70 nN and at RH about 30%) of deposited Ti substrate, APS coating, GO-APS coating and HRGO-APS coating under varied temperatures of 25 °C, 40 °C, 60 °C and 80 °C were shown in Figure 16. The friction force results were shown in Figure 17.

For the hydrophilic surfaces of the Ti substrate, APS coating and GO-APS coating, the adhesion and friction forces increased initially and then decreased as the testing temperature increased, as shown in Figure 16 and Figure 17. According to Frisbe [29], the AFM silicon probe tip would experience a tribochemical reaction to generate silicon hydroxyl groups in nano-tribological tests. When sliding against hydrophilic surface of Ti substrate, APS coating or GO-APS coating, the adhesion should be in the state of partial F_OH/OH_, which enhances the adhesion and friction forces in nano-tribology (as illustrated at Section 4.1.1). In light of the temperature raise, the tribochemical reaction was promoted and thus accelerate the hydroxylation of the silicon probe tip, which resulted in the increase of adhesion and friction forces of those three coatings. On the other hand, as the temperature continued to rise, the loss of water molecules owing to evaporation between contact surfaces weakened meniscus effect and tribochemical reaction, and thus resulted in reduction of the adhesion and friction forces. Those two factors played contradictory roles in the test, which can be conferred as the reason of the fluctuation of adhesion and friction forces of Ti substrate, APS coating or GO-APS coating in Figure 16 and Figure 17.

However for hydrophobic surface of HRGO-APS coating, the adhesion state between it and hydroxylated silicon probe was presumed to be F_OH/CHx_ which was much lower than the case of those three hydrophilic coatings. The water evaporation owing to temperature increase also reduced the adhesion of HRGO-APS coating. Moreover according to Zhao [33,34], as temperature raises, atomic thermal motion intensifies and thus the free movement room for atomics at the tribosliding interface increases, so that the consumed energy for tribosliding interface between probe tip and tested surface reduces. That factor was conferred to decrease the adhesion and friction forces of HRGO-GO APS coating.

According to the above nanofrictional investigation, the HRGO-APS coating performs more reliable and lower adhesion and friction forces under all circumstances, compared with Ti substrate, APS coating and GO-APS coating.

**Figure 18:**
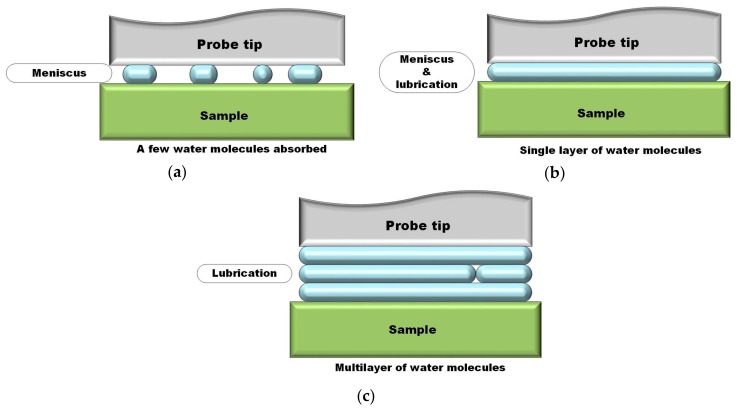
The effect of water molecules between contact surfaces in nanotribological tests.

The function of water molecules between the contact surfaces of probe tip and sample was illustrated in Figure 18. When a few water molecules were absorbed at low RH value (as in Figure 18a), the meniscus effect was the dominant factor. As RH increased, water molecules accumulated and a single layer or few layers of water molecules could be formed (as in Figure 18b). In that case, adhesion and friction forces were competitively affected by both water lubrication and water meniscus force. Finally, as RH increased to the highest value, multilayer of water molecules was formed between contact surfaces which resulted in the decrease of adhesion and friction forces for samples with hydrophilic surface (as in Figure 18c).

### 4.2. Nano wear Resistance of HRGO-APS Coating

**Figure 19:**
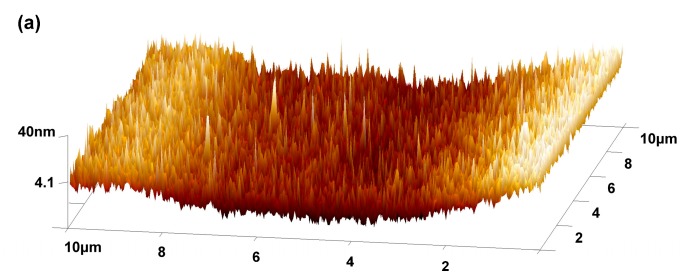

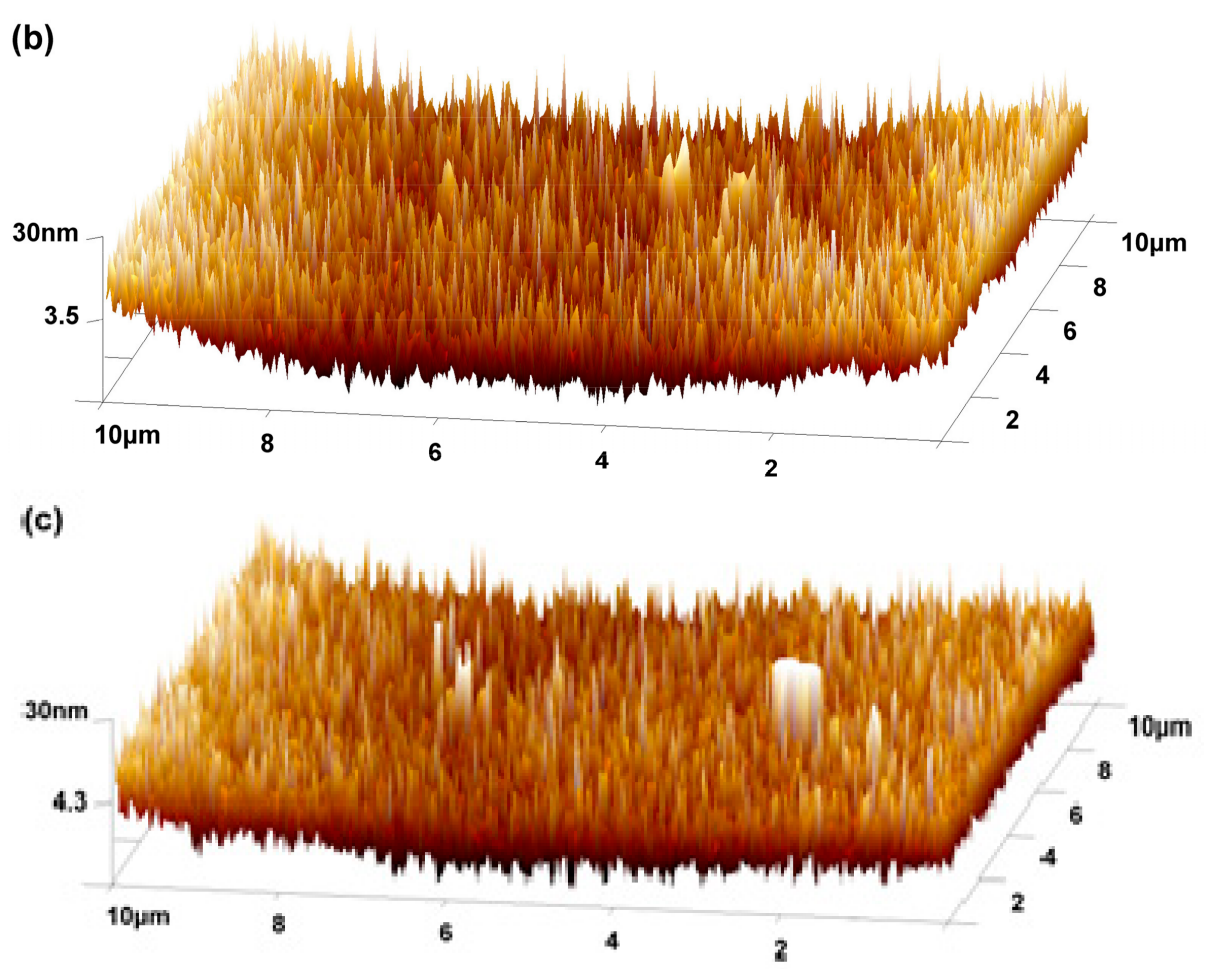
Wear scars of samples: (**a**) deposited Ti substrate, (**b**) GO-APS coating and (**c**) HRGO-APS coating.

A colloidal tip was used to mimic slide on the surface of HRGO-APS coating, GO-APS coating and the deposited Ti substrate by AFM under contact mode. The sliding rate was 2 Hz with a scanning length of 10 μm and applied loads of 2 μN, and the sliding duration for each sample was 5 s. The resultant AFM wear scar morphologies were detected by normal probe tips and shown in Figure 19.

After the wear test, the wear scar of Ti substrate (Figure 19a) was much more apparent than that of GO-APS (Figure 19b) and HRGO-APS coatings (Figure 19c). The results indicate that both GO-APS and HRGO-APS coatings can strengthen the wear resistance of the Ti substrate. It is roughly estimated that the depths of HRGO-APS, GO-APS both exceeded the thickness of corresponding coatings. Further wear scanning would be the wear of the substrate and thus was not investigated in this study. Nano wear volume can hardly detected owing to the rough surface of both tested area and the nearby area.

## 5. Conclusions

A HRGO-APS coating was deposited on titanium substrates via a self-assembly approach for lubricating and anti-wear applications. The process parameters of the GO coating deposition and its hydrothermal reduction were discussed, and the optimized process were: duration of GO coating for 12 h, hydrothermal reduction duration of 6–8 h, and temperature of ~135 °C. Then the nano-tribological behaviors were investigated under different applied loads, scanning speeds, relative humidity values and temperatures. The results showed that as-prepared HRGO-APS coating held lower and more reliable adhesion and friction forces under all tested circumstances. By the comparison test of nanowear performance of HRGO-APS coating, GO-APS coating and the deposited Ti substrate, the as-prepared HRGO-APS coating significantly improved the wear resistance of the substrate. Further efforts on new nanowear test design or testing equipment can be made in future studies for accurate determination of the wear volume of the coatings.

## Figures and Tables

**Table 1:** Variables of Equation (4) for Figure 12.

Variables	Ti	APS	GO-APS
A_1_	38.6	21.5	20.0
A_2_	47.7	26.6	29.0
x_0_	264.4	364.5	785.1
p	2.8	3.5	1.8

**Table 2:** Variables of Equation (4) for Figure 13.

Variables	Ti	APS	GO-APS
A_1_	3.9	2.6	1.9
A_2_	20.9	8.1	3.5
x_0_	292.1	397.7	226.3
p	1.5	2.4	1.6
